# Profiling of ribonucleotides and deoxyribonucleotides pools in response to DNA damage and repair induced by methyl methanesulfonate in cancer and normal cells

**DOI:** 10.18632/oncotarget.21521

**Published:** 2017-10-04

**Authors:** Jian-Ru Guo, Zheng Li, Cai-Yun Wang, Christopher Wai Kei Lam, Qian-Qian Chen, Wei-Jia Zhang, Vincent Kam Wai Wong, Mei-Cun Yao, Wei Zhang

**Affiliations:** ^1^ State Key Laboratory of Quality Research in Chinese Medicines, Macau Institute for Applied Research in Medicine and Health, Macau University of Science and Technology, Taipa, China; ^2^ School of Pharmaceutical Sciences, Sun Yat-Sen University, Guangzhou, China

**Keywords:** DNA damage, ribonucleotides, deoxyribonucleotides, perturbation, gene expression

## Abstract

The absolute and relative pool sizes of deoxyribonucleotides (dRNs) are essential in DNA replication fidelity, DNA damage and repair. We found in this study that although DNA damage induced by methyl methanesulfonate (MMS) seemed similar in cancer (HepG2) and normal (LO2) cells, more extensive alterations in ribonucleotides (RNs) and dRNs pools occurred in HepG2 cells indicating that HepG2 cells were more vigilant to DNA damage. After 10 h repair, RNs pools were still severely perturbed in LO2 cells. Compared to LO2 cells, deoxyribonucleotide triphosphates (dNTPs) pools in HepG2 cells elevated by more folds which could facilitate more efficient DNA repair and improve survival probability following DNA damage, although this should definitely lead to higher mutation rates. DNA repair was more efficient in HepG2 cells at S phase and it partly came to an end while DNA repair was still uncompleted in LO2 cells outside S phase. In conclusion, our results demonstrated that HepG2 and LO2 cells presented many differences in nucleotide metabolism, cell cycle checkpoints and DNA repair pathways in response to DNA damage, which could be potential targets for cancer treatment.

## INTRODUCTION

The integrity and stability of DNA are essential to each cell because DNA is the repository of genetic information, which is stored as a code consisted of deoxyribonucleotide triphosphates (dNTPs). However, DNA is constantly exposed to various environmental agents and numerous genotoxic chemicals, which can produce a large variety of DNA damages [1, 2]. The free dNTPs used in DNA synthesis are more susceptible to damage than are bases in duplex DNA [3].

To cope with these DNA damages, cells have developed a complex network of DNA repair mechanisms [1, 4]. A variety of different DNA repair pathways have been reported including direct reversal, base excision repair, nucleotide excision repair, mismatch repair, and recombination repair pathways. In these pathways, cells require extra amounts of dNTPs to repair DNA. In many cancer therapies, much focus has been put on damage directly to the DNA molecule. However, up-regulated DNA repair pathways can cause resistance to DNA-damaging chemotherapy and radiotherapy. So, manipulating DNA damage and repair has potential to sensitize cells to these therapies.

In the last few years, substantial progress has been made in the understanding of the biochemical mechanisms involved in DNA damage and repair. The cellular deoxyribonucleotides (dRNs) pools could present dramatically different alterations after diverse DNA-damaging treatment in different cells. Chabes et al. reported that DNA damage induced by 4-nitroquinoline-N-oxide (4-NQO) led to a 6 to 8 fold increase of dNTPs levels, which dramatically improved survival following DNA damage in in yeast cells [5]. However, for mutant yeast cells which fail to produce sufficient dNTPs upon DNA damage, their survival is compromised [6]. Compared with yeast, mammalian cells possess different mechanisms to regulate dNTPs perturbation caused by DNA damage [7]. It has been reported that underexpression of key enzymes involved in *de novo* deoxyribonucleoside biosynthesis and subsequent depletion of endogenous dNTPs pools partially caused DNA damage in human fibroblasts undergoing oncogene-induced senescence [8]. Furthermore, an exogenous supply of nucleosides to increase the dNTPs pools can reverse DNA damage and dramatically decreased oncogene-induced transformation [9]. As the significant role of dNTPs pools in DNA damage research, monitoring changes in the intracellular dNTPs pool could facilitate the investigations on mechanisms underlying DNA damage and repair.

Although many studies have contributed to the understanding of perturbation of dNTPs pools, the respective deoxyribonucleotide monophosphates (dNMPs) and diphosphates (dNDPs) have not been studied in DNA damage since their amounts are significantly lower than its respective triphosphate metabolites. Moreover, there is little knowledge about the difference between cancer and normal cells on up-regulation of dNTPs pools for DNA repair. As mutations is more extensively occurred in cancer cells than normal cells, the genetic modifications of nucleotide metabolism pathways or genetic defects in cancer may interfere or facilitate the alteration of the dNTPs pools in response to DNA damage. Thus, the elucidation of those differences can advance our understanding of the mechanisms behind the efficacy and toxicity of anticancer drugs.

To address these issues, the cellular ribonucleotides (RNs) and dRNs pools were determined in cancer (human hepatocellular cancer cell line, HepG2) and normal (human hepatocyte normal cell line, LO2) cells with or without methyl methanesulfonate (MMS) treatment that is known to cause DNA damage. Compared to LO2 cells, RNs and dRNs pools more extensively perturbed in HepG2 cells after DNA damage. After 10 h repair, RNs pools and dRNs proportions were nearly restored to normal levels in HepG2 cells, while RNs pools were still severely perturbed in LO2 cells. Moreover, dNTPs pools elevated more obviously in HepG2 cells, which could facilitate more efficient DNA repair and improve survival following DNA damage. Taken together, HepG2 cells repaired DNA damage mainly at S phase while LO2 cells performed DNA repair mainly at G1 and S phase, what's more, HepG2 cells succeed in DNA repair and survived from DNA damage while LO2 cells failed to repair DNA damage.

## RESULTS

### DNA damage detected by comet assay

**Figure 1:**
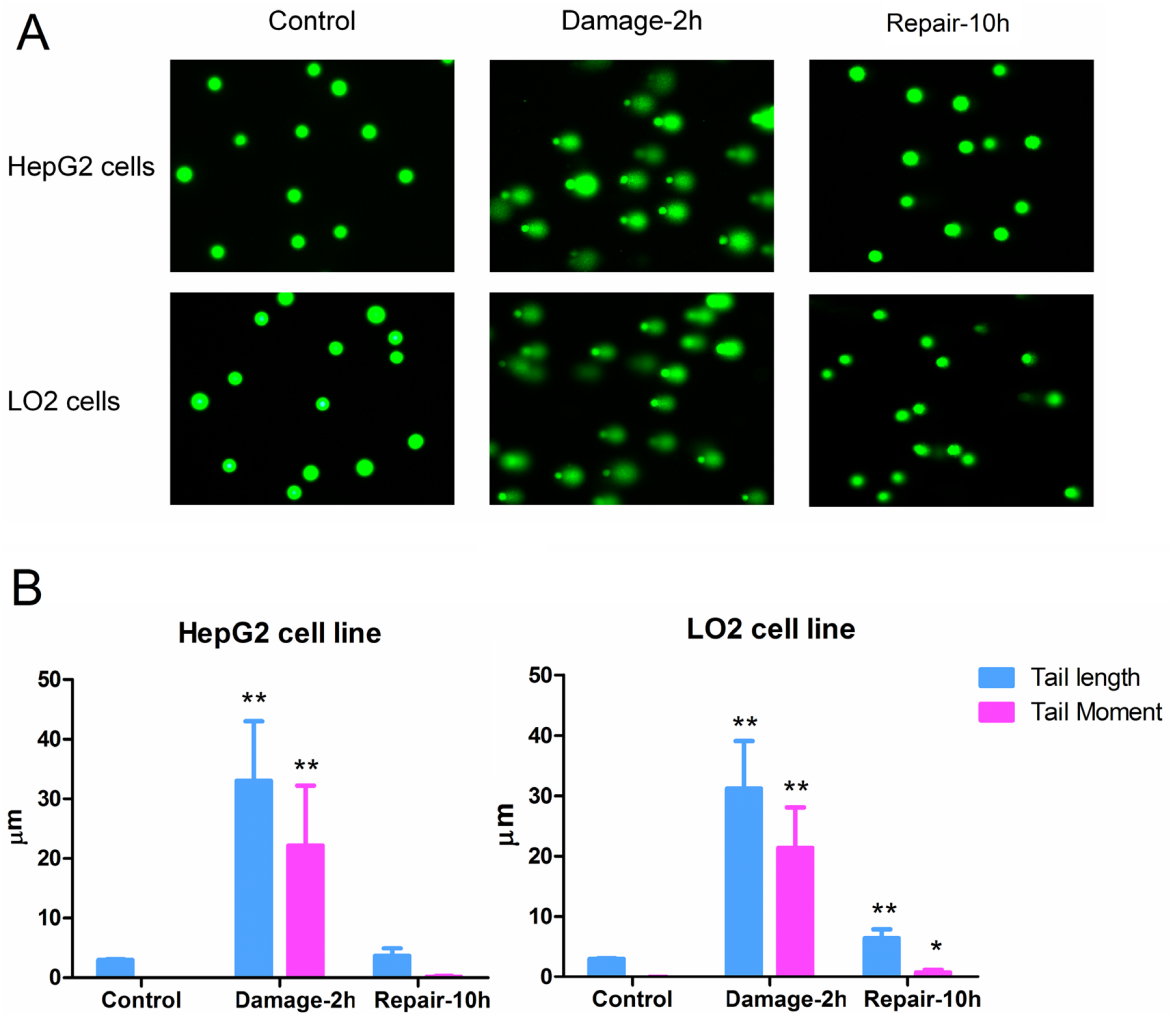
**(A)** DNA damage detected by comet assay. **(B)** Parameters for evaluation of DNA damage degree. (^*^ *P* < 0.05, ^**^ *P* <0.01, compared with the corresponding control group; ^#^ *P* <0.05, ^##^ *P* <0.01, compared with the corresponding damage group).

Based on the observed effects of MMS on cell viability, 1.0 mM MMS was chosen because it was the highest concentration that had no strong inhibitory effect on HepG2 and LO2 cells after 2 h incubation (cell viability > 85 % of control). To facilitate the analysis of DNA damage and repair, comet assays of HepG2 and LO2 cells with different incubation periods were performed. Compared with the control groups, longer tails in HepG2 and LO2 cells were seen after 2 h incubation with MMS. The tails were nearly back to normal after 10 h of recovery indicating the disappearance of double-strand breaks (DSBs) in the chromosomes of HepG2 and LO2 cells (Figure 1A). It was note-worthy that the tail levels of HepG2 and LO2 cells in the repair groups were different. Longer tail length and higher tail moment values were found in LO2 cells after 10 h recovery (Figure 1B).

### Multivariate statistical analysis of RNs and dRNs pools

**Figure 2:**
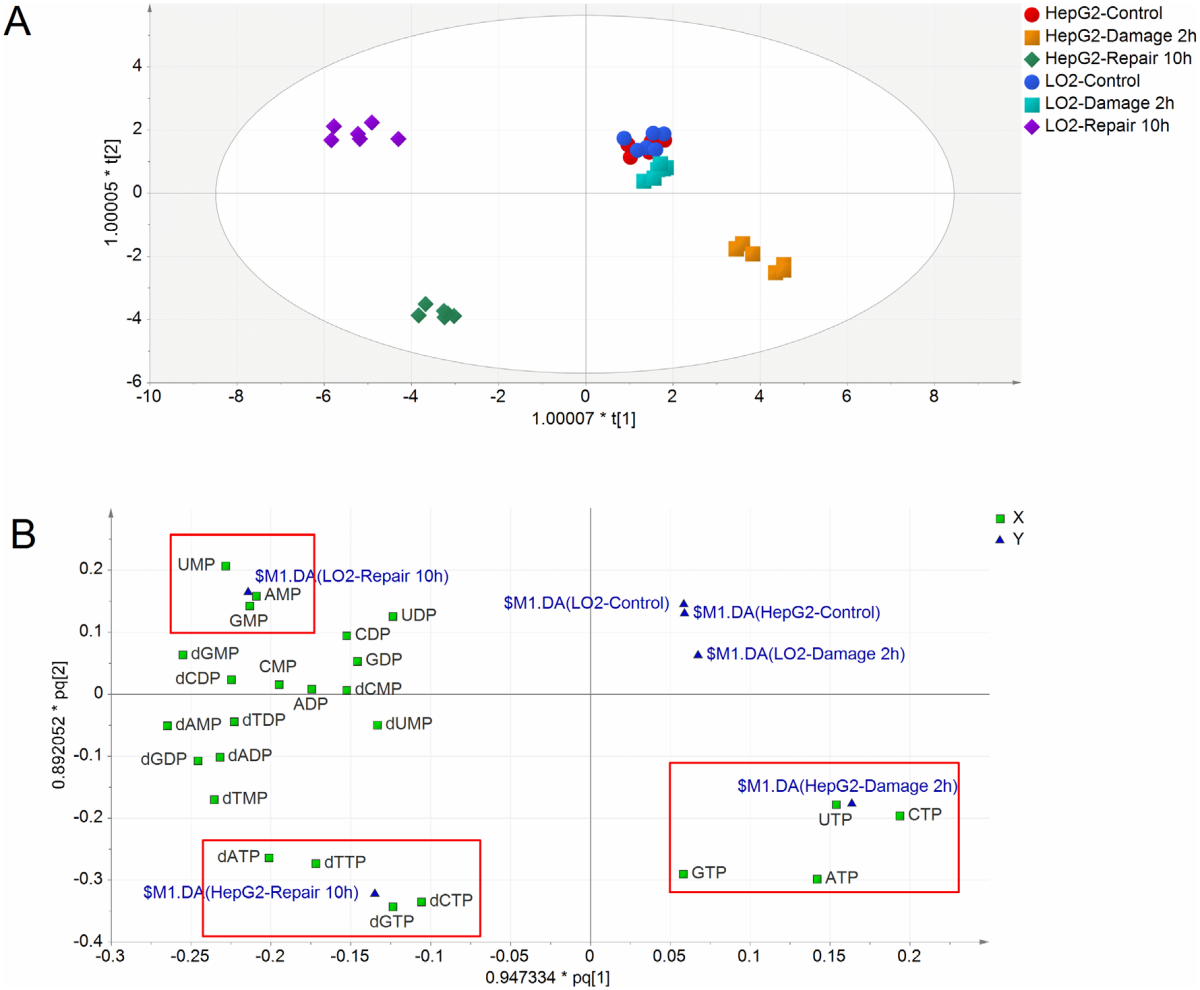
**(A)** Scores plot of OPLS-DA model for different groups (n=6). Scores plot describes the similarities between the Y-variables (groups) based on the X-variables (RNs and dRNs pools). **(B)** Loadings plot of OPLS-DA model for different groups. Loadings plot displays the relationship between RNs and dRNs pools and groups.

The levels of deoxyuridine triphosphate (dUTP), deoxyuridine diphosphate (dUDP) and deoxyuridine monophosphate (dUMP) are not shown in this paper since their levels were below the detect limit of the corresponding assays before and after MMS treatment. After quantitation of RNs and dRNs pool sizes, the absolute amount of each RNs and dRNs was used to obtain a data matrix consisting of 36 objects and 24 variables. Supervised orthogonal partial least squares discriminant analysis (OPLS-DA) model was constructed to understand and visualize the complex effect of MMS on RNs and dRNs pools using SIMCA-P version 14.0 (Umetrics Inc., Umeå, Sweden). The relative RNs and dRNs pools of each group (% of corresponding control) were used for data normalization of the two cell lines. As shown in Figure 2A, the control, damage and repair groups of HepG2 cells showed an appreciable separation based on the total alteration in the data. However, control and damage groups of LO2 cells could not get clear separation while the repair group achieved sufficient separation with the two groups. Thus, nucleotide metabolism altered sensitively in respond to DNA damage in HepG2 cells than LO2 cells. In addition, the RNs and dRNs pools varied differently between HepG2 and LO2 cells during DNA damage and repair (Figure 2B). DNA damage presented close association with increases in NTPs (ATP, GTP, CTP and UTP) pools, while DNA repair showed close relation with increases in dNTPs (dATP, dGTP, dCTP and dTTP) in HepG2 cells. However, DNA damage caused no clear alteration in RNs and dRNs pools and DNA repair showed close relation with increases in NMPs (AMP, GMP and UMP) pools in LO2 cells.

### Perturbation of RNs pools

As shown in Table 1, more extensive alterations in RNs pools occurred in HepG2 cells than LO2 cells after MMS treatment. Significant increases in NTPs pools were found in both HepG2 and LO2 cells. However, significant decreases in NDPs and NMPs (except CMP) were only observed in HepG2 cells, while no significant changes were found in almost all of these RNs in LO2 cells. Moreover, energy balance was severely perturbed only in HepG2 cells. AMP/ATP ratio, a key regulator of AMP-activated protein kinase (AMPK), was significantly decreased only in HepG2 cells. At some time, a noticeable elevation of energy charge, calculated by the following equation: energy charge = (ATP + 0.5 ADP)/(ATP + ADP + AMP), was observed only in HepG2 cells.

**Table 1:** RNs pools in HepG2 and LO2 cells of different groups (pmol/10^6^ cells)

	HepG2 cells			LO2 cells		
	Control	Damage-2h	Repair-10h	Control	Damage-2h	Repair-10h
ATP	9639.26±842.54	20316.44±1771.2^**^	13999.13±418.36^**##^	11649.62±1726.22	14991.59±1231.29^**^	10359.59±771.96^##^
ADP	645.12±98.28	517.22±86.69^*^	911.59±268.82^*##^	987.35±369.66	1069.25±356.93	1590.14±571.00^*^
AMP	922.66±185.67	227.71±48.76^**^	1001.31±461.64^##^	469.91±275.94	420.70±219.53	1498.73±491.19^**##^
CTP	1625.47±142.19	3285.95±362.83^**^	1907.51±121.91^**##^	1109.64±177.76	1795.88±175.22^**^	890.22±33.82^*##^
CDP	306.38±53.97	184.59±30.73^**^	373.05±158.45^#^	172.63±53.10	243.84±39.04^*^	251.18±11.69^**^
CMP	104.51±17.52	138.06±33.21^*^	166.82±17.50^**^	79.47±13.81	113.80±28.34^*^	197.40±51.28^**##^
GTP	2338.45±397.33	4539.54±500.33^**^	3901.29±752.82^**^	2011.69±316.66	3037.69±439.38^**^	2291.14±370.72^##^
GDP	150.36±19.25	107.06±11.83^**^	212.39±38.57^**##^	318.08±212.52	353.05±217.21	709.78±511.61
GMP	76.84±11.24	15.50±2.46^**^	100.62±15.05^**##^	31.33±18.47	34.46±19.76	126.89±48.75^**##^
UTP	2546.07±245.74	4872.52±845.87^**^	2997.31±794.52^##^	3960.81±2017.46	5209.07±2304.01	2465.55±965.87^#^
UDP	475.59±81.42	244.43±57.63^**^	472.62±238.24^#^	479.66±202.46	567.26±231.97	793.41±470.06
UMP	64.40±6.99	17.79±2.32^**^	60.81±21.53^##^	38.21±7.67	38.87±3.91	97.83±13.75^**##^
AMP/ATP	0.09±0.02	0.01±0^**^	0.07±0.03^##^	0.04±0.02	0.03±0.01	0.14±0.04^**##^
Energy charge	0.89±0.02	0.98±0.00^**^	0.91±0.03^##^	0.93±0.01	0.94±0.00^**^	0.83±0.01^**##^

^*^P < 0.05, ^**^P < 0.01, compared with corresponding control group; ^#^*P* < 0.05, ^##^ *P* < 0.01, compared with corresponding damage group.

Different alterations in RNs absolute amounts were found after 10 h repair between HepG2 and LO2 cells. RNs pools were restored to relative normal levels and energy imbalance disappeared in HepG2 cells. However, remarkable increases in NDPs, especially NMPs pools as well as noticeable down-regulation of energy metabolism were found in LO2 cells. These suggested that HepG2 cells might recover from DNA damage while LO2 cells might not have successfully recovered after 10 h repair.

### Perturbation of dRNs pools

Generally, dRNs pools in HepG2 cells were larger than these in LO2 cells (Table 2). Similar to perturbation of RNs pools, MMS treatment also caused more extensive alterations of dRNs pools in HepG2 cells that LO2 cells, especially dNDPs and dNMPs pools, which could support the above inference that HepG2 cells were more vigilant and quick in responding to DNA damage than LO2 cells. Significant increases in dRNs absolute amounts were found after 10 h repair in both HepG2 and LO2 cells. However, as compared with LO2 cells, HepG2 cells presented more fold increases in dNTPs pools, similar increases in dNDPs pools, and less fold increases in dNMPs pools. These results suggested that LO2 cells degraded dNDPs to dNMPs quickly, which is contradictory to HepG2 cells synthesizing dNTPs from dNDPs. As sufficient levels of dNTPs would facilitate DNA synthesis and subsequently DNA repair in general, this mechanism may reduce the efficiency of DNA repair but prevent high mutation rates in LO2 cells. On the other hand, HepG2 cells could undergo perform an efficient DNA repair mechanisms by increasing the dNTPs levels, although this could increase the mutation rates and replication errors.

**Table 2:** dRNs pools in HepG2 and LO2 cells of different groups (pmol/10^6^ cells)

	HepG2 cells			LO2 cells		
	Control	Damage-2h	Repair-10h	Control	Damage-2h	Repair-10h
dATP	37.87±4.35	36.16±5.38	126.89±10.92^**##^	16.82±3.28	11.13±1.31^**^	28.81±0.92^**##^
dADP	0.17±0.04	0.03±0.02^**^	0.63±0.24^**##^	0.04±0.03	0.02±0.02	0.14±0.06^**##^
dAMP	0.04±0.01	0.01±0^**^	0.16±0.05^**##^	0	0	0.02±0^**##^
dCTP	34.28±7.60	59.98±3.33^**^	100.07±10.16^**##^	16.9±6.66	22.93±7.20	24.63±6.94
dCDP	0.10±0.01	0.04±0.01^**^	0.24±0.09^**##^	0.02±0.02	0.03±0.02	0.08±0.03^**##^
dCMP	0.02±0.03	0.01±0.01	0.07±0.07	0	0	0
dGTP	25.49±4.69	57.20±10.52^**^	113.01±5.37^**##^	8.52±1.8	15.05±5.78^*^	16.69±4.08^**^
dGDP	2.95±0.25	1.61±0.41^**^	10.89±1.61^**##^	1.02±0.09	1.36±0.23^**^	3.34±1.40^**##^
dGMP	1.75±0.24	0.30±0.13^**^	7.50±3.10^**##^	0.09±0.05	0.16±0.11	0.86±0.17^**##^
dTTP	56.43±8.37	67.86±21.22	137.53±21.65^**##^	26.06±4.78	19.71±2.72^*^	38.38±1.45^**##^
dTDP	4.03±0.78	1.32±0.35^**^	8.90±4.42^*##^	1.68±0.58	1.36±0.25	3.43±0.93^**##^
dTMP	0.12±0.03	0.03±0.00^**^	0.40±0.01^**##^	0.03±0.01	0.03±0.00	0.05±0.01^**##^

^*^P < 0.05, ^**^P < 0.01, compared with the corresponding control group; ^#^*P* < 0.05, ^##^ *P* < 0.01, compared with the corresponding damage group.

### Perturbation of dNTPs pools

**Figure 3:**
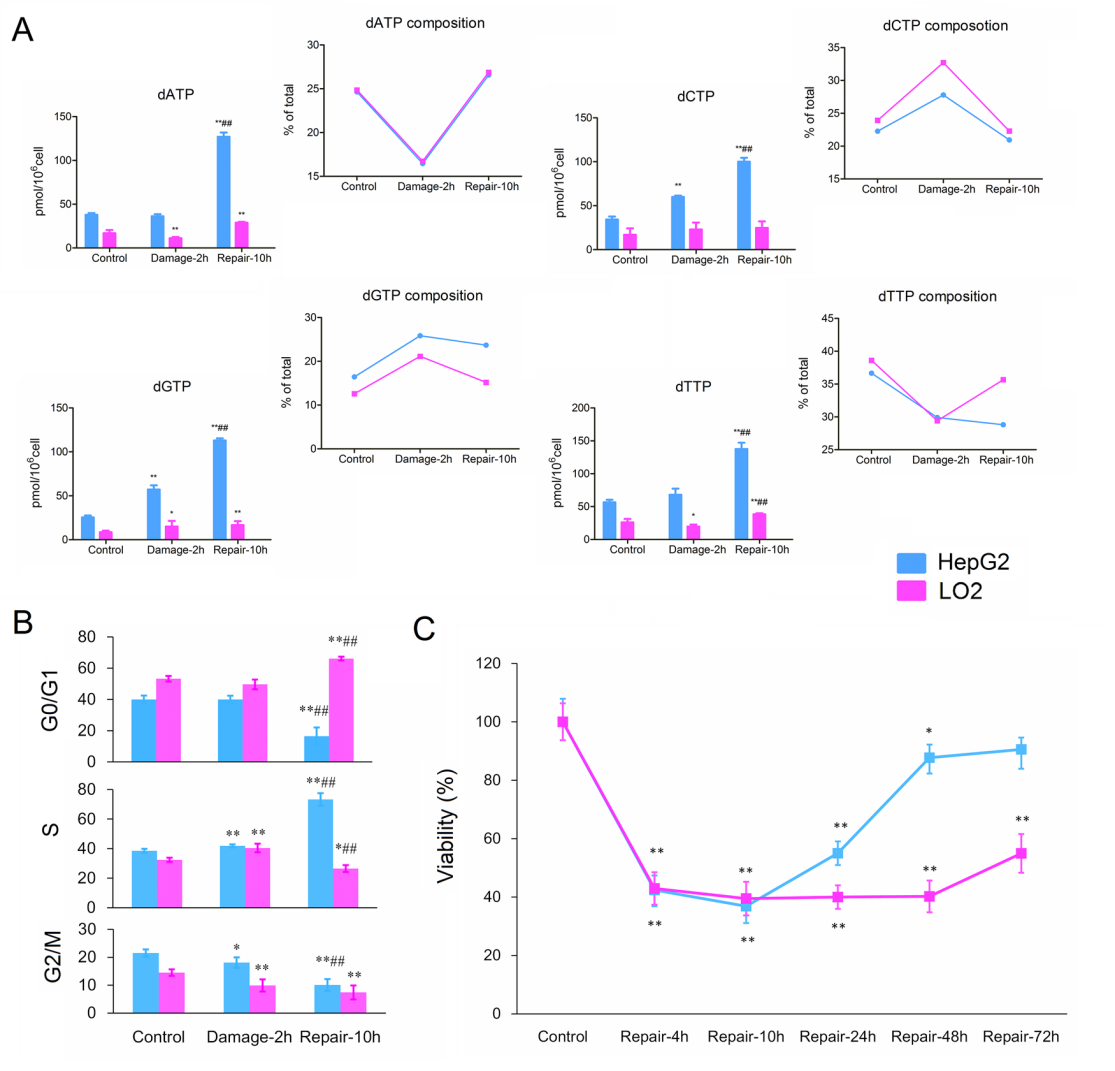
**(A)** Effects of MMS on dNTPs pools sizes in HepG2 and LO2 cells. **(B)** Cell cycle distribution of HepG2 and LO2 cells during DNA damage and repair. **(C)** Relative cell growth (% of control) at different repair point in time. Each data point is an average of two independent experiments (done in triplicate) and is reported as mean ± standard deviation. (^*^ *P* < 0.05, ^**^ *P* <0.01, compared with the corresponding control group; ^#^ *P* <0.05, ^##^ *P* <0.01, compared with the corresponding damage group).

Native dNTPs including dATP, dGTP, dCTP and dTTP have been quite tightly controlled under normal metabolic conditions. Without MMS treatment, the two cell lines shared similar dNTPs compositions, where dGTP, dCTP, dATP and dTTP accounted for about 15, 23, 25 and 37% of total dNTPs, respectively. As shown in Figure 3A, treatment of MMS resulted in increases in percentage of dCTP and dGTP from 22.27±4.34 to 27.79±3.74 and from 16.43±2.01 to 25.84±0.48 in HepG2 cells, and from 23.93±4.48 to 32.72±3.53 and from 12.6±1.41 to 21.14±3.09 in LO2 cells, respectively. Correspondingly, dATP and dTTP proportions exhibited similar decrease in HepG2 and LO2 cells after incubation with MMS. In addition, the absolute amounts of dCTP and dGTP were greater than that of the control cells without MMS in HepG2 and LO2 cells. HepG2 cells presented no remarkable changes in dATP and dTTP levels, while dATP and dTTP in LO2 cells showed a similar decrease after incubation of MMS. Compared with response of yeast, DNA damage induced by MMS led to quite different changes in dNTPs pools of mammalian cells.

It was noteworthy that the proportions of four dNTPs in LO2 cells returned to normal group levels, while the dGTP proportion was still up-regulated and dTTP proportion was still down-regulated in HepG2 cells after 10 h recovery. Although, imbalances of the four dNTPs pools have genotoxic consequences, dGTP accumulation was more strongly mutagenic than that of others [10]. In addition, dNTPs absolute amounts presented about 3-fold increases in HepG2 cells compared to those in the control group, while they showed only about 1.5-fold increases in LO2 cells compared to corresponding control group.

### Perturbation of dNDPs and dNMPs pools

Different from LO2 cells presenting no unified alteration in dNDPs pools, significant decreases in dNDPs pools were found in HepG2 cells after MMS treatment. However, there were about 3-fold increases in dNDPs pools in LO2 cells after 10 h recovery, which is similar to the trend observed in HepG2 cells. Similar to dNDPs, dNMPs pools presented significant decreases in HepG2 cells while no unified alteration in LO2 cells after MMS treatment. During DNA repair, dNMPs showed about 3-fold increases in HepG2 cells, while up to 6-fold increases in LO2 cells. Because the accumulations of dNDPs could not subsequently lead to increased levels of dNTPs in LO2 cells, this evidence suggests that dNDPs were mainly degradated to dNMPs in LO2 cells while dNDPs were catalyzed to dNTPs in HepG2 cells.

### Cell cycle distribution during DNA damage and repair

In order to response to DNA damage, cell division is arrested in many eukaryotic cells, which lead to perturbation in dNTPs pool sizes. As shown in Figure 3B, the cell cycle distributions of HepG2 and LO2 cells in respective control group (2 h) presented obvious difference, while changed with the same trend after DNA damage. The percentage of HepG2 and LO2 cells in S phase both increased after incubation with MMS. Nevertheless, a big difference between HepG2 and LO2 cells was observed in the cell division after 10 h repair. During DNA repair, LO2 was found cell cycle arrest at G0/G1 phase increasing from 53.2±1.8 % (control group) to 66.1±5.6 %. In contrast to LO2 cells, HepG2 cells increased significantly the percentage of cells in the S phase from 38.5±1.4 % (control group) to 73.3±2.3 %. Usually, the concentration of dNTPs is highest in S phase in normal condition, so these results were consistent with the changes of dNTPs pools in the two cell lines after 10 h repair.

### Cell growth during DNA repair

The relative cell growth results showed significant differences between HepG2 and LO2 following serum stimulation for 2 days (Figure 3C). Owing to cell cycle arrest for DNA repair after DNA damage, the two cell lines showed similar recovery rates at 4- and 10-h time points. However, the relative cell number of HepG2 increased from 36.88±4.14% (10-hour recovery) to 87.72±4.46 % (48-hour recovery), while LO2 kept unchanged between 39.50±5.77 % (10-hour recovery) and 40.22±5.43 % (48-hour recovery). Obviously, cancer (HepG2) cells could more successfully survive from DNA damage induced by MMS than normal (LO2) cells. Thus, first priority for carcinoma cell may be survived, while the mutation rate and authenticity of the DNA has been taken into account by the normal cells.

### PCR array for DNA damage and repair

**Figure 4:**
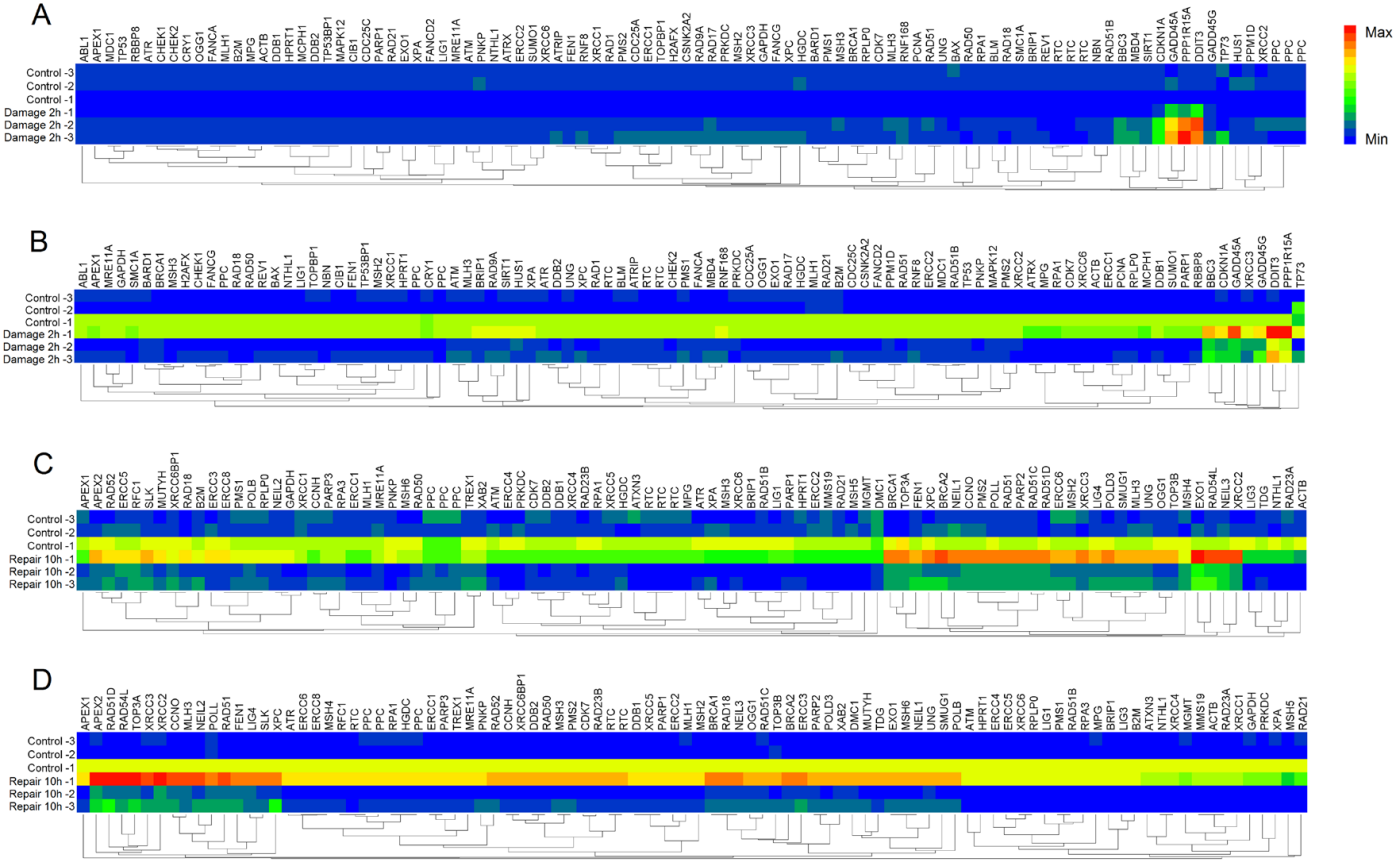
Heat maps of differentially expressed genes analyzed by DNA Damage and DNA Repair Signaling Pathway RT2 Profiler PCR Array **(A)** DNA Damage Array for HepG2 cells. **(B)** DNA Damage Array for LO2 cells. **(C)** DNA Repair Array for HepG2 cells. **(D)** DNA Repair Array for LO2 cells. Each column represents a single gene and each row represents a single group. Expression levels are colored blue for low intensities and red for high intensities.

**Figure 5:**
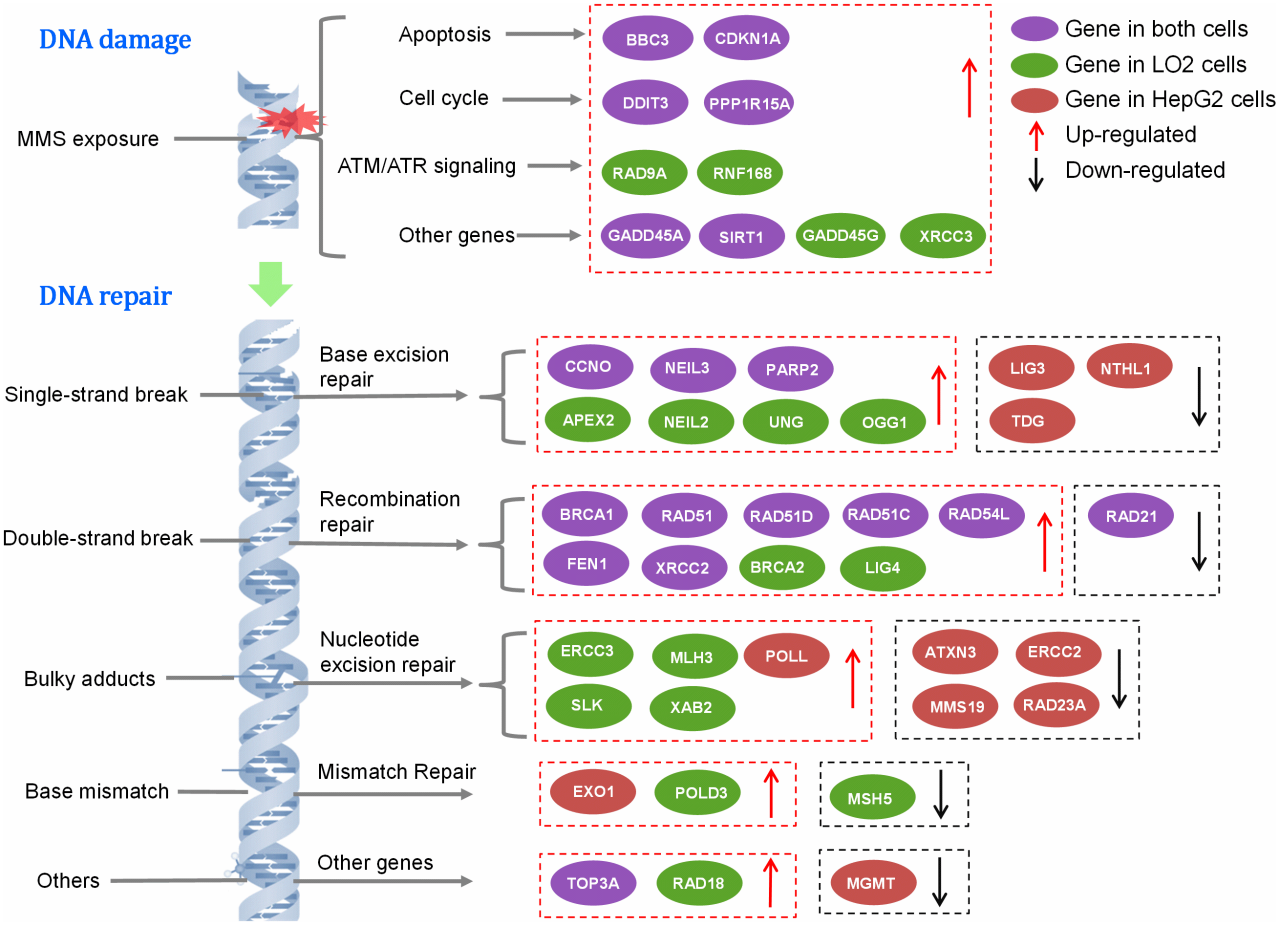
The signaling pathways of DNA damage and repair genes

Gene expression profile of DNA damage and repair was contrastively investigated in HepG2 and LO2 cells. The heat maps provided a visualization of the selected groups for every gene. Hierarchical clustering of obtained genes was performed, which separates the samples into their corresponding phenotype groups (Figure 4). Clustering indicated that most of the genes tested separated into several broad clusters in each group. The tested genes with high fold changes binded to each other, clustered closely. Relatively high expression values are shown in red while blue indicates low values. Among these genes, which was considered to be significant up- or down-regulated based on expression changes (≧1.5-fold) and *p* value <0.05. The details of altered genes in DNA damage and repair were listed in Table 3. As shown in Figure 5, during DNA damage, upregulated DDIT3 and PPPIR15A in HepG2 and LO2 cells were cell cycle controlling genes, which supported the results of cell cycle arrest analysis. Obvious differences between HepG2 and LO2 cells were RAD9A and RNF168 involved in ATM/ATR signaling, which might partly explain the subsequent difference in cell cycle distribution between the two cell lines during DNA repair.

**Table 3:** The list of altered genes in DNA damage and repair signaling pathway (fold change >1, indicated gene up-regulation; fold change <1, indicated gene down-regulation)

Cell line	Gene (fold change) in DNA damage				Gene (fold change) in DNA repair			
HepG2	**PPP1R15A** (7.40)	**DDIT3** (7.33)	**GADD45A** (5.82)	**CDKN1A** (2.62)	**EXO1** (2.89)	**RAD54L** (2.51)	**NEIL3** (2.20)	**XRCC2** (1.97)
	**BBC3** (1.80)	**SIRT1** (1.50)			**TOP3A** (1.96)	**BRCA1** (1.83)	**FEN1** (1.68)	**RAD51D** (1.66)
					**PARP2** (1.62)	**RAD51** (1.59)	**POLL** (1.55)	**RAD51C** (1.53)
					**CCNO** (1.52)			
					**RAD23A** (0.45)	**MGMT** (0.50)	**NTHL1** (0.53)	**LIG3** (0.57)
					**ERCC2** (0.59)	**RAD21** (0.61)	**ATXN3** (0.64)	**MMS19** (0.64)
					**TDG** (0.66)			
LO2	**DDIT3** (9.75)	**PPP1R15A** (7.58)	**GADD45A** (3.49)	**BBC3** (3.20)	**APEX2** (2.94)	**RAD51D** (2.77)	**TOP3A** (2.67)	**RAD54L** (2.52)
	**GADD45G** (2.98)	**CDKN1A** (2.12)	**XRCC3** (1.90)	**RAD9A** (1.65)	**XRCC2** (2.43)	**FEN1** (2.18)	**RAD51** (2.12)	**CCNO** (1.99)
	**SIRT1** (1.54)	**RNF168** (1.52)			**MLH3** (1.94)	**NEIL2** (1.94)	**LIG4** (1.88)	**RAD18** (1.86)
					**BRCA2** (1.82)	**BRCA1** (1.81)	**SLK** (1.81)	**NEIL3** (1.80)
					**OGG1** (1.58)	**RAD51C** (1.58)	**PARP2** (1.56)	**POLD3** (1.54)
					**UNG** (1.54)	**XAB2** (1.54)		
					**RAD21** (0.53)	**MSH5** (0.58)		

After 10 h repair, many genes were still up-regulated. They were mainly involved in base excision repair (BER), homologous recombination repair (HRR) and nucleotide excision repair (NER). The two cell lines shared many altered genes implicated in BER and HRR. However, HepG2 cells showed much more down-regulated genes and LO2 cells presented much more up-regulated genes required in DNA repair, which suggested that DNA repair partly came to an end in HepG2 cells while it was still uncompleted in LO2 cells.

## DISCUSSION

### Perturbation of RNs and dRNs pools with DNA damage and repair

Endogenous RNs and dRNs pools play essential roles in a broad range of key cellular functions. dRNs are synthesized from corresponding RNs. An unbalanced change of RN and dRN pool sizes can lead to genetic abnormalities or cell death in mammalian cells. The synthesis of dNTPs is highly regulated and is important for DNA replication and repair in all living cells [10], because levels that are too high or too low can easily lead to increased rates of mutagenesis and promotion of cancer development [11].

Studies conducted in yeasts and mammalian cells report that imbalance of endogenous dNTPs pools can lead to DNA damage, inhibition of DNA replication fork progression, cell-cycle arrest, growth retardation, high mutation rates, and carcinogenesis via several interrelated mechanisms [8, 12, 13]. The most obvious mechanism is that dNTPs depletion slows processivity or even completely arrests DNA polymerase. After MMS treatment, more extensive alterations in RNs and dRNs pools occurred in HepG2 cells and energy balance was severely perturbed only in HepG2 cells. These results indicated that HepG2 cells were more vigilant and quick to take response to DNA damage than LO2 cells. After 10 h repair, RNs pools and dRNs proportions were restored to relative normal levels and energy imbalance was disappeared in HepG2 cells. Moreover, all dRNs (dNTPs, dNDPs and dNMPs) pools increased in parallel in HepG2 cells. As *de novo* DNA synthesis is required to fill the gaps generated in the course of DNA repair [14], when dNTPs levels are restored, DNA replication can resume and fork stalling appear to be eliminated. Furthermore, it was reported that increased dNTPs levels in yeast leads to improved resistance to DNA damage [15, 16], which is primarily repaired by nucleotide excision repair (NER). However, over-compensated high levels of dNTPs could lead to genome instability [17]. Thus, HepG2 cells could perform efficient DNA repair facilitated by increased dNTPs pools, although this could increase the mutation rates and replication errors. In summary, HepG2 cells could recover from DNA damage and initiate cell division benefiting from increased dNTPs pools.

However, remarkable increases in NDPs and NMPs pools and noticeable down-regulation of energy metabolism were found in LO2 cells after 10 h repair. The increases in the cellular AMP:ATP and ADP:ATP ratios could activate AMPK to induce a p53-dependent G1 cell-cycle arrest [18], which was supported by the cell cycle arrest at G1 phase in LO2 cells. Furthermore, the accumulation of dNDPs in LO2 cells is prone to degrade to dNMPs rather than to synthesize dNTPs. This mechanism may reduce the efficiency of DNA repair but prevent the high mutation rates. Therefore, these results suggested that LO2 cells might not successfully recover from DNA damage after 10 h repair.

We believed that more efficient DNA synthesis in cancer cells could improve survival following DNA damage, although this definitely leads to higher mutation rates. It was further supported by the relative cell growth results of HepG2 and LO2 cells following serum stimulation for 2 days. The relative cell number of HepG2 cells presented progressive fold increase, while LO2 cells kept unchanged. In brief, we believed that cancer (HepG2) cells were more vigilant to DNA damage and efficient in DNA repair than normal (LO2) cells after MMS treatment.

### Cell division with DNA damage and repair

In normal physiological condition, cellular dNTPs pool sizes fluctuate during the cell cycle, which is connected to the expression of enzymes for dNTP synthesis, particularly ribonucleotide reductase (RR) [10, 19]. The RR is responsible for the reduction of NDPs to their corresponding dNDPs and sequently dNTPs, which are then used in the synthesis of DNA during replication or DNA repair [20]. Human RR is composed of three known subunits, RRM1, RRM2 and P53R2 that are differentially regulated during the cell cycle [21, 22]. RRM1 expression is constitutive, and the RRM1 protein is metabolically stable [23], whereas levels of the RRM2 protein rise and fall, with the highest levels during S phase. Thus, cycling cells present high dNTPs pools at S phase and low dNTPs pools outside S phase.

In response to DNA damage, cells trigger multifaceted responses such as cell cycle arrest, DNA repair, or apoptosis [24]. The DNA-damage checkpoint is the mechanism that detects damaged DNA and generates a signal that arrests cells in the G1 phase, S phase (DNA synthesis), or G2 phase, and induces the transcription of repair genes. The position of arrest within the cell cycle varies depending upon the phase in which the damage is sensed [25]. Several studies have reported that cells are most sensitive to MMS during S phase [26–28]. Exposure to MMS brought about accumulation of cells in S phase, especially early S phase. Therefore both HepG2 and LO2 cells respond to DNA damage by arresting the cell cycle at S phase. However, a significant difference between HepG2 and LO2 cells was observed in the cell division after 10 h recovery. The cell cycle arrest at S phase continuously increased in HepG2 cells while the cell cycle arrest at G0/G1 phase occurred in LO2 cells. This was accordant with the difference of dNTPs pools between the two cell lines.

The complete nuclear DNA replication is replicated only in cycling cells during S-phase [29], whereas cycling and quiescent cells replicate mitochondrial DNA and repair damaged DNA during their whole existence. Thus, cycling cells require a large supply of dNTPs during S-phase while outside S-phase cells consume much smaller amounts of dNTPs. In this study, after 10 h recovery, HepG2 cells mainly distributed in S phase, and in which dNTPs pools were greatly elevated. Furthermore, based on the results of cell growth during 2 days after DNA damage, cell proliferation was initiated in HepG2 cells after 10 h recovery. While LO2 cells stopped dividing and remained in G0, and they may stay in this state for some time before they start dividing again or even stay in G0 permanently until die. These results indicated that HepG2 cells could efficiently repair damaged DNA mainly at S phase, while LO2 cells could tardily perform DNA repair outside of S phase. Therefore, targeting the S checkpoint is particularly attractive for cancer therapy because loss of G1 check-point control is a common feature of cancer cells, making them more reliant on the S checkpoint to prevent DNA damage triggering cell death.

### Gene expression with DNA damage and repair

MMS is a monofunctional DNA alkylating agent and a known carcinogen [30, 31] and primarily methylates DNA on N^7^-deoxyguanine and N^3^-deoxyadenine [32]. Although the N^7^-methylguanine adduct may be nontoxic and nonmutagenic, N^3^-methyladenine is a lethal lesion that inhibits DNA synthesis and needs to be actively repaired [31, 33]. Recognition of these DNA lesions starts a protein cascade, which finally results in cell cycle arrest (checkpoint activation) and DNA repair. The three pathways responsible for the removal of most N3-methyladenine lesions are bypass repair (or post-replication repair), recombination repair, and base excision repair [34]. If DNA repair fails, or is over-whelmed by to many DNA lesions, these sensors initiate DNA damage can result in apoptosis.

DNA damage caused up-regulation of many genes involved in cell cycle arrest, apoptosis and others in HepG2 and LO2 cells. Likewise, large-scale genetic alterations were also detected in HepG2 and LO2 cells during DNA repair. The two cell lines shared many altered genes implicated in BER and HRR which indicate that BER and HRR are two functional DNA repair pathways commonly used in both HepG2 and LO2 cells. As the principal mechanism of DSBs repair, HRR is generally restricted to S phase because it uses sister-chromatid sequences as the template to mediate faithful repair. This could be the reason for main up-regulated genes belong to HRR pathway and HepG2 cells could perform efficient DNA repair at S phase. More down-regulated genes and less up-regulated genes were found in HepG2 cells than LO2 cells indicating that HepG2 cells were more rapid and successful in DNA repair than LO2 cells.

In this study, cancer (HepG2) and normal (LO2) cells presented significantly differences in DNA damage responses. DNA damage caused more extensive alterations of RNs and dRNs pools in HepG2 cells than LO2 cells indicating that HepG2 cells were more vigilant and quick to respond to DNA damage than LO2 cells. After 10 h repair, RNs pools and dNTPs proportions were nearly restored to normal levels in HepG2 cells. HepG2 cells presented more obvious increases of dNTPs pools than LO2 cells, which concurred with the cell cycle at S phase in HepG2 cells and G1 phase in LO2 cells. This is because cycling cells required a large supply of dNTPs for DNA replication during S-phase while cells outside the S-phase consumed much smaller amounts of dNTPs. The significant increases in cellular AMP:ATP and ADP:ATP ratios in LO2 cells could contribute to cell cycle arrest at G1 phase. The increases of dNTPs pools in HepG2 cells could facilitate more efficient DNA repair and improve survival following DNA damage, although this definitely leads to higher mutation rates. Based on gene expression, HepG2 cells were more efficient in DNA repair by HRR and BER at S phase and DNA repair partly came to an end in HepG2 cells while it was still uncompleted in LO2 cells. Cancer cells usually present genetic instability and significant difference of DNA damage response compared with normal cells. Taken together, our results demonstrated that HepG2 and LO2 cells presented many differences in dNTPs metabolism, cell cycle checkpoints and DNA repair pathways to respond to DNA damage, which could be interesting prospect for this cancer treatment.

## MATERIALS AND METHODS

### Chemicals and reagents

LC-MS grade methanol, acetonitrile and acetic acid were purchased from Anaqua Chemical Supply (Houston, TX, USA). Trioctylamine, 1, 1, 2-trichlorotrifluoroethane, methyl methanesulfonate (MMS), dimethyl sulfoxide (DMSO) and stable isotope labeled adenosine-^13^C10^15^, N5-triphosphate (ATP^13^C^15^,N) were purchased from Sigma Aldrich Chemical Co., St. Louis, MO, USA. Ultra-pure water was obtained from a Milli-Q Gradient water system (Millipore, Bedford, MA, USA). Dulbecco's Modified Eagle Medium (DMEM), RPMI Medium 1640, penicillin–streptomycin solution, fetal bovine serum (FBS) and SYBR^®^ safe DNA gel stain were obtained were obtained from Invitrogen Corp., CA, USA. Ethylenediaminetetraacetic acid disodium salt dehydrate (EDTA-2Na) and Triton^®^ X-100 were bought from MP Biomedicals, LLC., Illkirch, France. Human hepatocellular cancer cell line (HepG2) was bought from American Type Culture Collection (ATCC; Rockville, MD, USA). Human hepatocyte normal cell line (LO2) was obtained from type Culture Collection of Chinese Academy of Sciences (Shanghai, China).

### Cell culture and MTT assay

HepG2 cells were cultured in DMEM medium and LO2 cells were maintained in RPMI 1640 medium containing 10 % (*v*/*v*) FBS, 100 units/mL penicillin, 100 μg/mL streptomycin in a 37°C humidified incubator with a 5% CO_2_ atmosphere. HepG2 and LO2 cells were seeded in 96-well plates at 1×10^4^ cells/well. After 24 h from seeding, they were exposed to a range of concentrations of MMS (0.25, 0.5, 1.0, 1.5, 2.0, 4.0, 6.0, 8.0 and 10.0 mM) for different periods (1, 2, 3, 4 and 5 h). MTT assay was performed by a microplate UV/VIS spectrophotometer (Infinite M200 PRO, Tecan Austria GmbH 5082, Grödig, Austria). The cell number was determined using a hemocytometer.

### MMS induced DNA damage and repair

HepG2 and LO2 cells were seeded at a density of 5 × 10^5^ cells/well in 6-well plates and incubated for 24 h. Both HepG2 and LO2 cells were divided into different groups as follows: cells that were incubated with medium alone in control group, and cells that were exposed to 1.0 mM MMS for 2 h in damage group. After 2 h MMS treatment, cells in repair group were washed thoroughly to remove MMS and further incubated with fresh medium for 10 h to repair DNA damage. Subsequently, DNA damage was investigated following the “Comet assay” procedure.

### Comet assay

In this study, MMS induced DNA damage was measured by alkaline comet assay according to previously published methods [35]. Comets were detected by a fluorescence microscope (Leica Microsystems Ltd., Wetzlar, Germany). Images were analyzed by comet assay IV software (Globalebio Ltd., Beijing, China) according to the method presented by Helma et al [36]. Tail length and tail moment were used to evaluate the degree of DNA damage.

### Determination of RNs and dRNs

Monolayer cells were washed with ice-cold PBS once and were trypsinized with pancreatin. Cells from two or three dishes were then re-suspended with 12 mL ice-cold phosphate buffered isotonic saline solution (PBS). Then cell number was counted before centrifugation at 1,000 rpm for 5 minutes. And the cell pellet was washed with 1 mL ice-cold PBS again and spun down at 1,000 rpm for 5 minutes. It was then treated with 150 μL of 15% trichloroacetic acid (TCA) containing 7.5 μL of 20.0 μM ATP^13^C^15^,N as internal standard and placed on ice for 10 minutes. After centrifugation at 13,500 rpm for 15 min in the cold room, the acidic supernatant was separated and neutralized twice with 80 μL mixture of trioctylamine and 1, 1, 2-trichlorotrifluoroethane (a volume ratio of 45 to 55). RNs and dRNs pools were determined based on the method previously described [37]. The cellular concentrations of nucleotides were calculated by dividing the absolute amount of each RN and dRN in each sample by the corresponding cell number.

### Cell cycle analysis

Cells were harvested and fixed in 70% (*v*/*v*) cold ethanol at 4°C overnight. The fixed cells were collected by centrifugation and re-suspended in PI/RNase Staining Buffer (Cell Cycle Detection Kit, KeyGEN BioTECH, NanJing, China) for staining DNA, and finally analyzed on a FACSAriaTM III flow cytometer (BD Biosciences, NJ, US).

### Cell growth assay

HepG2 and LO2 cells were seeded in 96-well plates at 1 × 10^4^ cells/well following the “Cell culture” procedure. After incubation with 1.0 mM MMS for 2 h, cells were washed thoroughly to remove MMS, and further incubated with fresh medium for 4, 10, 24, 48 and 72 h. Cell viability was determined via the MTT assay.

### DNA damage and repair RT^2^ PCR array

The human DNA Damage and Repair PCR array were used to evaluate potential alterations of related genes during MMS-induced DNA damage and repair. Both DNA Damage and Repair PCR Array comprised 84 genes involved in DNA damage and repair signaling pathways. Total RNA from cell lines was extracted with the RNeasy Microkit (Qiagen, Hilden, Germany) according to the manufacturer's protocol. Then total RNA was reverse-transcripted using the RT^2^ First Strand Kit (Qiagen, Hilden, Germany). 84 key genes from damage and repair pathway were simultaneously assayed with the RT^2^Profiler PCR array plate (cat. no. 30430 and 30430, Qiagen, Hilden, Germany) on ABI 7500 (Applied Biosystems, CA, USA). All samples were analyzed in triplicate and data analysis was performed using the Qiagen's integrated web-based software package for the PCR Array System.
